# Supplementary material for: Adjusting for truncated study duration in recurrent event analysis: A weighting approach for clinical trials
Source: PLoS One. 2026 Jan 7;21(1):e0339887. doi: 10.1371/journal.pone.0339887 (PMC12779063; doi:10.1371/journal.pone.0339887)
Supplement: S1 File — (DOCX) [file pone.0339887.s001.docx]

R Codes for Simulation

library(survival)

library(tidyverse)

library(kableExtra)

library(flexsurv)

library(tidyr)

library(reshape2)

library(coxphw)

library(epiDisplay)

library(dplyr)

finalfunction <- function(N,variation_visits,proportion_probability,lambda, rho, beta,prob_status){

### Simulation function

simulRecEv <- function(n,n.j, lambda, rho, beta,prob_status){

# covariate --> N Bernoulli trials

x <- rbinom(n,1,0.3)

#if(x==1){x1=rbinom(n,1,0.8)}

#if(x==0){x1=rbinom(n,1,0.3)}

x.ij=matrix(cbind(rep(x,n.j)),nrow=n.j,ncol=n)

y.ij<-matrix(nrow=n.j,ncol=n) # Creating covariate

for (j in 1:n){

for (i in 1:n.j){

value_x=x.ij[i,j]

if(value_x==1){y.ij[i,j]=rbinom(1,1,0.8)}

if(value_x==0){y.ij[i,j]=rbinom(1,1,0.3)}

}

}

###keeping same x for

for (ja in 1:n.j){

print(ja)

x.ij[ja,]=x.ij[1,]

y.ij[ja,]=y.ij[1,]

}

v.ij<-matrix(nrow=n.j,ncol=n) # Creating covariate

for (j in 1:n){

for (i in 1:n.j){

value_x=x.ij[i,j]

value_y=y.ij[i,j]

if(value_x==0 & value_y==0){v.ij[i,j]=runif(1,0,1)}

if(value_x==0 & value_y==1){v.ij[i,j]=runif(1,0,1)}

if(value_x==1 & value_y==0){v.ij[i,j]=runif(1,0,1)}

if(value_x==1 & value_y==1){v.ij[i,j]=runif(1,0,1)}

}

}

# Exponential latent event times T1-T3

# Exponential latent event times T1-T3

t.ij<-matrix(nrow=n.j,ncol=n) # Creating time Variable

for (j in 1:n){

for (i in 1:n.j){

t.ij[i,j]<- (- log(runif(1,0,1)) / (lambda * exp(x.ij[i,j] * beta)))

t.ij[i,j]=t.ij[i,j]+20

if (t.ij[i,j]<28)

{

for (try in 1:100)

{

t.ij[i,j]<- (- log(runif(1,0,1)) / (lambda * exp(x.ij[i,j] * beta)))

t.ij[i,j]=t.ij[i,j]+20

if (t.ij[i,j]>=28){break}

}

t.ij[i,j]=t.ij[i,j]

}

}

}

status<-matrix(0,nrow=n.j,ncol=n) # Creating status Variable

for (j in 1:n){

for (i in 1: n.j){

status[i,j]<- rbinom(1,1,prob=prob_status)

}

}

data <- data.frame(t(status),t(t.ij),t(v.ij),t(x.ij),t(y.ij))

}

#### looop for variation in n and n.j

variation_visits=variation_visits

visits=sample(variation_visits, size = N, prob = proportion_probability, replace = TRUE)

visits_1=data.frame(table(visits))

list_n=as.numeric(as.character(visits_1$Freq))

list_n.j=as.numeric(as.character(visits_1$visits))

empty_dataframe=data.frame()

### variying the n and n.j

for(i in 1:length(variation_visits)){

n=list_n[i]

n.j=list_n.j[i]

## calling the first function inside the function

data <- simulRecEv(n=n,n.j=n.j, lambda=Lambda, rho=Rho, beta=TrueBeta,prob=prob_status)

##variation in column names based on n and n.j names

T_name=c()

for (k in 1:n.j){

T_name=append(T_name,c((rep(paste0("T",k),1)),(rep(paste0("S",k),1)),

(rep(paste0("X",k),1)),(rep(paste0("V",k),1)),(rep(paste0("Y",k),1))))

}

T_name=sort(T_name)

names(data) = c(T_name)

### binding data for all choices of n and n.j

if (i==1) {new_empty=data}

if (i!=1) {new_empty=merge(new_empty,data,all = TRUE)}

}

new_empty$id=seq(1,N,1)

return(new_empty)

}

modelfunction <- function(OUTPIUTS){

####subset only required column names

OUTPIUTS_1=OUTPIUTS[c("id","X1","T1","T2","T3","T4","T5","T6","T7","T8","T9","S1","S2","S3","S4","S5","S6","S7","S8","S9")]

colnames(OUTPIUTS_1)=c("id","X1","T_1","T_2","T_3","T_4","T_5","T_6","T_7","T_8","T_9","S_1","S_2","S_3","S_4","S_5","S_6","S_7","S_8","S_9")

##two different data frame

OUTPIUTS_1_Time=OUTPIUTS_1[c("id","X1","T_1","T_2","T_3","T_4","T_5","T_6","T_7","T_8","T_9")]

OUTPIUTS_1_Status=OUTPIUTS_1[c("id","S_1","S_2","S_3","S_4","S_5","S_6","S_7","S_8","S_9")]

##wide to long and merge

data_long_time=melt(OUTPIUTS_1_Time,id.vars = c("id","X1"))

colnames(data_long_time)=c("id","X","F","T")

data_long_status=melt(OUTPIUTS_1_Status,id.vars = "id")

colnames(data_long_status)=c("id_1","FS","S")

final_data=cbind(data_long_time,data_long_status)

###final_data

final_data1=final_data

final_data1=final_data1[final_data1$T>0,]

#final_data_last_time <- aggregate(final_data1$T, by = list(final_data1$id), FUN = tail, n = 1)

final_data_last_time <- final_data1 %>%

group_by(id) %>%

summarize(last_value = tail(T, n = 1))

final_data_last_time=data.frame(na.omit(final_data_last_time))

final_data_time_sum <- aggregate(final_data1$T, by = list(final_data1$id), FUN = sum, n = 1)

final_data_time=final_data_time_sum-final_data_last_time

final_data_time$Id=rownames(final_data_time)

final_data_time$time_last=final_data_last_time$last_value

final_data_time$Total_time=final_data_time_sum$`sum.final_data1$T`

final_data_time=final_data_time[,c(2:5)]

colnames(final_data_time)=c("sum_time_n-1","ID","time_n","sum_time_n")

E=quantile(final_data_time$sum_time_n,0.2)

E

Weight_denom=final_data_time$time_n/(E-final_data_time$`sum_time_n-1`)

Weight_denom[Weight_denom>1] <- 1

Weight_denom[Weight_denom<0] <- 1

Weight=1/Weight_denom

final_data_time$Weight=Weight

###

No_of_visits=data.frame(table(final_data1$id))

colnames(No_of_visits)=c("ID","Max_visit")

#Added no of visits

final_data_new=merge.data.frame(final_data,No_of_visits,by.x = "id",by.y = "ID")

#Added weight

final_data_new_1=merge.data.frame(final_data_new,final_data_time[,c("ID","Weight")],by.x = "id",by.y = "ID")

##order columns

final_data_new_1=final_data_new_1[order(final_data_new_1$id,final_data_new_1$F),]

final_data_new_1$new=1

sumss=final_data_new_1 %>% group_by(id) %>% mutate(tSTOP = cumsum(new))

final_data_new_1$visit=sumss$tSTOP

#final weight

final_data_new_1$w <- ifelse(final_data_new_1$Max_visit ==final_data_new_1$visit, final_data_new_1$Weight, 1)

##factor for F

final_data=final_data[c("id","X", "F","T","S")]

final_data$F=as.factor(final_data$F)

##creating new variable lag and cum sum

final_data=final_data[order(final_data$F),]

final_data=final_data[order(final_data$id),]

data_obs_long=final_data %>% group_by(id) %>% mutate(tSTOP = cumsum(T))

data_obs_long <- data_obs_long %>%group_by(id) %>% mutate(t_start = lag(tSTOP,1)) %>% relocate(tSTOP, .before = tSTOP)

data_obs_long$t_start[which(is.na(data_obs_long$t_start))] <- 0

#data_obs_long$T[which(is.na(data_obs_long$T))] <- 0

#data_obs_long$S[which(is.na(data_obs_long$S))] <- 0

data_obs_long=data_obs_long %>% group_by(id) %>% mutate(Cstatus = cumsum(S))

data_obs_long$Tstart_GT=0

data_obs_long$tSTOP_GT=data_obs_long$T

data_obs_long=data_obs_long[!is.na(data_obs_long$tSTOP_GT),]

###added weights in the final data

data_obs_long=merge.data.frame(data_obs_long,final_data_new_1[,c("id","F","w")],by.x = c("id","F"),by.y = c("id","F"))

return(data_obs_long)

}

model_fianal = function(N,variation_visits,proportion_probability,lambda=Lambda, rho=Rho, beta=TrueBeta,prob_status=prob_status){

###Output simulated dataframe

MB=0

MB_WT=0

BM=0

BM_WT=0

SE=0

SE_WT=0

CI_L=0

CI_U=0

CI_WT_L=0

CI_WT_U=0

time_data_out=data.frame(matrix(NA, nrow = 1000, ncol = 100))

for (i in 1:1000)

{

OUTPIUTS=finalfunction(N,variation_visits,proportion_probability,lambda=Lambda, rho=Rho, beta=TrueBeta,prob_status=prob_status)

time_data=OUTPIUTS[,c("T1","T2","T3","T4","T5","T6","T7","T8","T9")]

time_data$Total_length=rowSums(time_data[,c("T1","T2","T3","T4","T5","T6","T7","T8","T9")],na.rm = TRUE)

time_data_out[,i]=time_data$Total_length

# if(i==1)

# {

# print("first data")

# }

data_obs_long=modelfunction(OUTPIUTS)

#PWP_GT model

PWP_GT <- coxph (Surv(Tstart_GT,tSTOP_GT,S)~X + cluster(id)+strata (Cstatus),data = data_obs_long)

#PWP_GT_WT <- coxphw (Surv(Tstart_GT,tSTOP_GT,S)~X + cluster(id)+strata (Cstatus),data = data_obs_long,caseweights=data_obs_long$w)

PWP_GT_WT <- coxph (Surv(Tstart_GT,tSTOP_GT,S)~X + cluster(id)+strata (Cstatus),data = data_obs_long,weights=w)

beta <- summary(PWP_GT)$coefficient[1]

#beta_WT <- coef(PWP_GT_WT)[1]

beta_WT <- summary(PWP_GT_WT)$coefficient[1]

# print(i)

# print(beta)

# print(beta_WT)

MB[i]=beta

MB_WT[i]=beta_WT

BM[i]=beta-TrueBeta

BM_WT[i]=beta_WT-TrueBeta

##SE Normal

SE[i]=summary(PWP_GT)$coefficient[3]

CI_L[i] = beta - 1.96*SE[i]

CI_U[i] = beta + 1.96*SE[i]

##se weighted

SE_WT[i]=summary(PWP_GT_WT)$coefficient[3]

CI_WT_L[i] = beta_WT - 1.96*SE_WT[i]

CI_WT_U[i] = beta_WT + 1.96*SE_WT[i]

}

Model_estimates <- data.frame(MB,MB_WT,BM,BM_WT,CI_L,CI_U,CI_WT_L,CI_WT_U)

Model_estimates[which(Model_estimates$CI_L<=TrueBeta & Model_estimates$CI_U>=TrueBeta),"CP"] = 1

Model_estimates[which(Model_estimates$CI_L<=TrueBeta & Model_estimates$CI_U<=TrueBeta),"CP"] = 0

Model_estimates[which(Model_estimates$CI_L>=TrueBeta & Model_estimates$CI_U>=TrueBeta),"CP"] = 0

Model_estimates[which(Model_estimates$CI_WT_L<=TrueBeta & Model_estimates$CI_WT_U>=TrueBeta),"CP_WT"] = 1

Model_estimates[which(Model_estimates$CI_WT_L<=TrueBeta & Model_estimates$CI_WT_U<=TrueBeta),"CP_WT"] = 0

Model_estimates[which(Model_estimates$CI_WT_L>=TrueBeta & Model_estimates$CI_WT_U>=TrueBeta),"CP_WT"] = 0

return(Model_estimates)

}

## Scenario 1 (3 to 9 visits)

N=1000 #total number of individuals

variation_visits=c(3,4,5,6,7,8,9) # no of visits

proportion_probability=c(0.1,0.1,0.2,0.1,0.1,0.2, 0.2) # proportion of total N distributed for visits

Lambda <- 1

Rho <- 0.1

TrueBeta <- 3

prob_status <- 0.2

model_esti_S1=model_fianal(N,variation_visits,proportion_probability,lambda=Lambda, rho=Rho, beta=TrueBeta,prob_status=prob_status)

summ(model_esti_S1)

ci(model_esti_S1$MB)

ci(model_esti_S1$MB_WT)

## Scenario 2 (4 to 9 visits)

N=1000 #total number of individuals

variation_visits=c(4,5,6,7,8,9) # no of visits

proportion_probability=c(0.1,0.2,0.1,0.2,0.2, 0.2) # proportion of total N distributed for visits

Lambda <- 1

Rho <- 0.1

TrueBeta <- 3

prob_status <- 0.2

model_esti_S2=model_fianal(N,variation_visits,proportion_probability,lambda=Lambda, rho=Rho, beta=TrueBeta,prob_status=prob_status)

summ(model_esti_S2)

ci(model_esti_S2$MB)

ci(model_esti_S2$MB_WT)

## Scenario 3 (5 to 9 visits)

N=1000 #total number of individuals

variation_visits=c(5,6,7,8,9) # no of visits

proportion_probability=c(0.2,0.1,0.2,0.3,0.2) # proportion of total N distributed for visits

Lambda <- 1

Rho <- 0.1

TrueBeta <- 3

prob_status <- 0.2

model_esti_S3=model_fianal(N,variation_visits,proportion_probability,lambda=Lambda, rho=Rho, beta=TrueBeta,prob_status=prob_status)

summ(model_esti_S3)

ci(model_esti_S3$MB)

ci(model_esti_S3$MB_WT)

## Scenario 4 (6 to 9 visits)

N=1000 #total number of individuals

variation_visits=c(6,7,8,9) # no of visits

proportion_probability=c(0.2,0.3,0.3,0.2) # proportion of total N distributed for visits

Lambda <- 1

Rho <- 0.1

TrueBeta <- 3

prob_status <- 0.2

model_esti_S4=model_fianal(N,variation_visits,proportion_probability,lambda=Lambda, rho=Rho, beta=TrueBeta,prob_status=prob_status)

summ(model_esti_S4)

ci(model_esti_S4$MB)

ci(model_esti_S4$MB_WT)
